# Lead poisoning due to gunshot bullet in contact with cerebrospinal fluid: case report

**DOI:** 10.1590/S1516-31802009000100011

**Published:** 2009-05-11

**Authors:** Paulo Roberto de Madureira, Eduardo Mello De Capitani, Ronan José Vieira, Alice Momoyo Sakuma, Adriana Safioti Toledo, Suely Moreira Mello

**Affiliations:** 1 MD, PhD. Assistant professor, Poisoning Control Center, School of Medical Sciences, Hospital das Clínicas, Universidade Estadual de Campinas (Unicamp), Campinas, São Paulo, Brazil.; 2 PhD. Head, Atomic Absorption Laboratory, Instituto Adolfo Lutz, São Paulo, Brazil.; 3 MSc. Nurse, Poisoning Control Center, School of Medical Sciences, Hospital das Clínicas, Universidade Estadual de Campinas (Unicamp), Campinas, São Paulo, Brazil.; 4 MSc. Poisoning Center Laboratory, Poisoning Control Center, School of Medical Sciences, Hospital das Clínicas, Universidade Estadual de Campinas (Unicamp), Campinas, São Paulo, Brazil.

**Keywords:** Lead poisoning, Wounds, gunshot, Cerebrospinal fluid, Chelation therapy, Aminolevulinic acid, Intoxicação por chumbo, Ferimentos por arma de fogo, Líquido cefalorraquidiano, Terapia por quelação, Ácido aminolevulínico

## Abstract

**CONTEXT:**

Lead poisoning due to retained gunshot bullets is a well-known clinical problem that is fairly frequently described in the literature. The risk factors for this occurrence relate mainly to whether the lead bullet is in contact with the joint fluid or cerebrospinal fluid (CSF). The treatment for these cases entails chelation therapy while symptoms are shown and definitive surgical removal of the bullet as a potential source of lead. The aim of this paper is to describe a clinical case of lead poisoning due to a retained gunshot bullet in contact with CSF.

**CASE REPORT:**

A 42-year-old male was hit by gunshot bullets during a holdup, and one of them was retained in the spinal cord. Six years later, he developed intense low back pain and underwent laminectomy. Nine years later, he then underwent arthrodesis on L5-S1, but he developed intense abdominal pain after the surgical procedure. For five years, he was treated with calcium versenate in five-day cycles, with a good response. The chelation therapy cycles showed great efficacy during symptomatic periods, thus reducing the symptoms and signs of poisoning and promoting great amounts of lead excretion, thereby reducing the total lead burden responsible for the symptoms. Fortunately, over the last four years, the symptoms have improved and the urine levels of aminolevulinic acid (ALA) have declined, to reach complete normalization. This shows that a healing process is probably taking place on the spinal wound, thereby isolating the bullet fragments from CSF contact.

## RESUMO

**CONTEXTO:**

A intoxicação por chumbo devida a projétil retido em ferimento por arma de fogo é uma complicação já conhecida e descrita na literatura. O risco de intoxicação endógena por chumbo está associado ao contato do projétil com o líquido sinovial ou líquido o cefalorraquidiano. O tratamento requer terapia de quelação e retirada cirúrgica do projétil como tratamento definitivo. Este artigo descreve caso clínico de paciente que desenvolveu intoxicação por chumbo devida a projétil retido em contato com líquido cefalorraquidiano.

**RELATO DE CASO:**

Paciente masculino, 42 anos, foi baleado durante assalto e teve projéteis que se alojaram no abdômen, perna direita e coluna lombo-sacra. Seis anos depois, desenvolveu intensa lombociatalgia e foi submetido a laminectomia. Nove anos após o acidente, foi submetido a artrodese de L5-S1, quando foi tentada a retirada do projétil, sem sucesso, desenvolvendo no pós-operatório intensa dor abdominal. Foi então feito diagnóstico de intoxicação por chumbo, que foi tratada com gluconato de cálcio, com boa resposta. Durante os cinco anos seguintes, fez ciclos de quelação com ácido etilenodiaminotetracético (EDTA) cálcico, com boa evolução. Os ciclos de quelação mostraram grande eficácia na redução dos sinais e sintomas da intoxicação, promovendo um grande aumento da excreção de chumbo e reduzindo a carga corpórea total de chumbo responsável pelos sintomas. Nos últimos quatro anos, apresentou melhora dos sintomas de intoxicação, com diminuição dos níveis de ALA urinário até a normalização, mostrando que provavelmente houve um processo de cicatrização da lesão, isolando os fragmentos de chumbo do contato com o líquor.

## INTRODUCTION

Lead poisoning due to retained gunshot bullets is a well-known clinical problem that is fairly frequently described in the literature.^1^^,^^2^^,^^3^ The risk factors for this occurrence relate mainly to whether the lead bullet is in contact with the joint fluid or cerebrospinal fluid (CSF). The acidic pH of these fluids promotes lead solubilization, with mobilization into plasma, and consequent action on target organs. The time that elapses between the accident and the initial symptoms ranges from three months to an indefinite time, according to the bullet area that is in contact with organic fluids. The treatment for these cases entails chelation therapy while symptoms are shown and definitive surgical removal of the bullet as a potential source of lead.^4^ The diagnosis of lead poisoning can be confirmed by blood lead assaying or by measuring some of the indirect parameters for the effect of lead on hemoglobin synthesis, like delta-aminolevulinic acid (ALA) in blood or urine, zinc protoporphyrin or free protoporphyrin.^4^

The aim of this paper is to describe a clinical case of lead poisoning caused by a retained gunshot bullet in contact with CSF. Table 1 shows the results from systematic searching for similar studies using the PubMed, Literatura Latino-Americana e do Caribe em Ciências da Saúde (Lilacs) and Cochrane databases. Among the studies found, the most significant ones have been discussed and cited below.

**Table 1.** Results from systematic search in scientific literature databases for studies similar to the present case report

PubMed database	
*MeSH major topic and key words*	Number of studies from 1962 to 2008
“lead poisoning” and “gunshot wound”	92
“lead poisoning” and “gunshot wound” and “human”	81
“lead poisoning” and “gunshot wound” and “human” and “case reports”	58
“lead poisoning” and “gunshot wound” and “human” and “chelation therapy”	12
“lead poisoning” and “gunshot wound” and “central nervous system”	0
“lead poisoning” and “gunshot wound” and “spinal fluid”	0
Lilacs database	
*DeCS major topic and DeCS key words*	
“intoxicação por chumbo” (and) “ferimentos por arma de fogo”	6
Cochrane Library data-base	
*Search MeSH major topic and key words*	
“lead poisoning” and “gunshot wound”	1

MeSH = Medical Subject Headings; Lilacs = Literatura Latino-Americana e do Caribe em Ciências da Saúde; DeCS = Descritores em Ciências da Saúde.

## CASE REPORT

**Figure 1.**
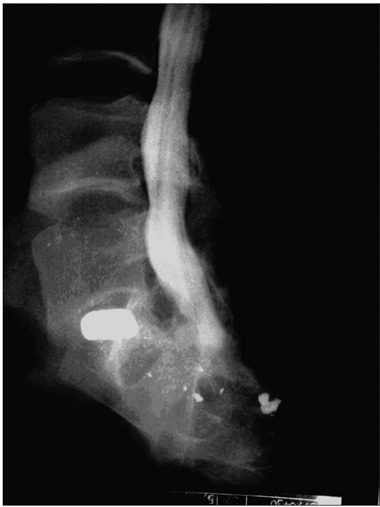
Lateral view of the bullet close to the spinal cord canal.

CAJP, a 42-year-old male real estate businessman, was hit by gunshot bullets in 1992 during a holdup. Bullets were found in the abdomen and right leg, and laparotomy was needed in order to perform resection on a segment of the small intestine. Another bullet was retained close to the spinal cord (Figure 1).

Six years later, he developed intense lumbar back pain, and the physicians attending the case realized that the source of pain was probably the retained bullet, which was lodged between L5 and S1. At that time, he underwent laminectomy in an attempt to remove the bullet. However, this operation was unsuccessful and caused the bullet to fragment.

**Figure 2.**
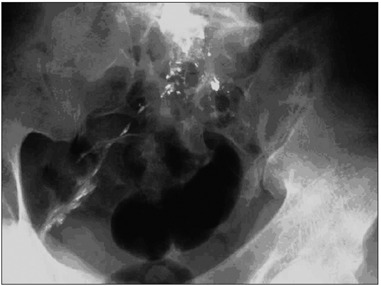
Lead precipitation from the bullet, throughout the sciatic sheath.

Nine years after the accident, the patient had his first episode of abdominal pain. He then underwent arthrodesis on L5-S1, but he developed intense abdominal pain after the surgical procedure. Radiographs produced at this time, two days after the surgical procedure, showed remarkable dispersion of lead from the bullet, with lead precipitation throughout the sciatic nerve sheath (Figure 2).

He was then treated with calcium versenate intravenously^4^ for five days, with a good response. ALA in urine was measured as 122 mg/l (reference value, RV = 4.5 mg/l) before this chelation therapy, and as 8.9 mg/l after the therapy. Total 24-hour lead in urine (PbU24h) over the five days of therapy was 47,623 mg, thus showing an outstanding level of lead excretion. Two weeks later, he showed recurrence of symptoms, with ALA of 55 mg/l. A new cycle of calcium versenate was then prescribed, with a good symptomatic response and a high level of lead excretion (26,679 mg).

**Figure 3.**
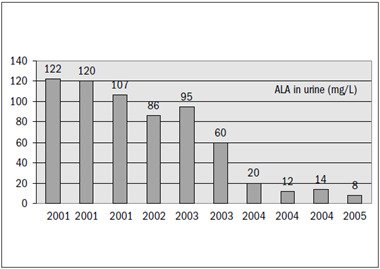
Aminolevulinic acid (ALA) in urine (mg/l) over the years of treatment and follow-up. No chelation therapy was necessary after 2003.

Over the next three months, he remained well. Then, in August 2001, the symptoms recurred and ALA in urine was measured as 120 mg/l, and a new therapy cycle was administered, using ethylenediaminetetraacetic calcium disodium (EDTACaNa_2_). From this last chelation cycle onwards, he remained less symptomatic, necessitating one more cycle during 2001, another in 2002 and two more cycles in 2003, when he started to be asymptomatic. His ALA in urine decreased progressively to normal values during 2004 and 2005 (Figure 3). Over the last two years (2006 and 2007), he remained asymptomatic and he has not shown up for examination since then.

## DISCUSSION

Scuderi et al. screened 12 patients with a bullet or bullet fragments within the intervertebral disk space, for evidence of lead poisoning. Only one of them showed clinical evidence of lead poisoning, and this patient underwent partial laminectomy and diskectomy, with excision of the bullet fragments. The patient’s complaints resolved two months after the operation.^1^ Grogan and Bucholz described another patient who developed acute lead poisoning 12 years after a gunshot wound and who continued to present a bullet lodged between the third and fourth lumbar vertebrae. This patient underwent three courses of chelation therapy, and the symptoms and blood lead levels started to decline only after laminectomy, with removal of the bullet and excision of an anterior cystic lesion formed by the presence of the bullet fragment.^5^

The surgical procedure usually provides preventive and definite treatment,^1^^,^^5^ but in the present case it seems to have been of little benefit. On the contrary, it seems to have put fragments of the bullet into contact with CSF, and consequently mobilized lead into the blood circulation, thus promoting lead poisoning symptoms for the first time in his clinical history since the accident.

The chelation therapy cycles showed great efficacy during symptomatic periods. They reduced the symptoms and signs of poisoning and promoted great amounts of lead excretion, thereby reducing the total lead burden responsible for the symptoms, as documented by the decreasing levels of ALA in urine over the last four years. The complete normalization of ALA levels shows that the bullet fragments probably somehow became isolated from the CSF (perhaps by fibrotic scar tissue formation around them), or were chemically transformed into insoluble salts.

Since then, the symptoms have subsided, and new cycles of EDTACaNa_2_ have not been considered necessary so far.

## References

[1] Scuderi GJ, Vaccaro AR, Fitzhenry LN, Greenberg S, Eismont F (2004). Long-term clinical manifestations of retained bullet fragments within the intervertebral disk space. J Spinal Disord Tech.

[2] de Madureira PR, De Capitani EM, Vieira RJ (2000). Lead poisoning after gunshot wound. Sao Paulo Med J.

[3] Janzen DL, Tirman PF, Rabassa AE, Kumar S (1995). Lead “bursogram” and focal synovitis secondary to a retained intrarticular bullet fragment. Skeletal Radiol.

[4] De Capitani EM, Azevedo FA, Chasin AAM (2003). Diagnóstico e tratamento de intoxicações. Metais: gerenciamento da toxicidade.

[5] Grogan DP, Bucholz RW (1981). Acute lead intoxication from a bullet in an intervertebral disc space. A case report. J Bone Joint Surg Am.

